# Creating a sustainable action-oriented engagement infrastructure—a UMN-MIDB perspective

**DOI:** 10.3389/fnint.2022.1060896

**Published:** 2022-12-15

**Authors:** Anita C. Randolph, Alex Henry, Amy Hewitt, Angie P. Mejia, Raj Sethuraju, Meriah DeJoseph, Melissa Koenig, Jed T. Elison, Damien A. Fair

**Affiliations:** ^1^Masonic Institute for the Developing Brain, University of Minnesota, Minneapolis, MN, United States; ^2^Department of Pediatrics, University of Minnesota, Minneapolis, MN, United States; ^3^Institute on Community Integration, University of Minnesota, Minneapolis, MN, United States; ^4^Center of Learning Innovation, University of Minnesota—Rochester, Rochester, NY, United States; ^5^School of Law Enforcement and Criminal Justice, Metropolitan State University, Saint Paul, MN, United States; ^6^Institute of Child Development, University of Minnesota, Minneapolis, MN, United States

**Keywords:** community engagement, diversity, infrastructure building, diversifying STEAM, neuroscience, inequities in education

## Abstract

Following the murder of George Floyd on May 25, 2020, Minneapolis represented the epicenter of protests that would reverberate internationally and re-instantiate a reckoning of the systemic and institutional racism that plagues American society. Also in the summer of 2020, and after several years of planning, the University of Minnesota (UMN) launched the Masonic Institute for the Developing Brain (MIDB), an interdisciplinary clinical and community research enterprise designed to create knowledge and engage all members of our community. In what follows, we describe the mission of the MIDB Community Engagement and Education (CEEd) Core and adjacent efforts within the UMN neuroscience and psychology community. Inherent to these efforts is the explicit attempt to de-center the dominant academic voice and affirm knowledge creation is augmented by diverse voices within and outside of traditional academic institutions. We describe several initiatives, including the Neuroscience Opportunities for Discovery and Equity (NODE) network, the NextGen Psych Scholars Program (NPSP), the Young Scientist Program, among others as exemplars of our approach. Developing and fortifying sustainable pathways for authentic community-academic partnerships are of central importance to enhance mutually beneficial scientific discovery. We posit that traditional academic approaches to community engagement to benefit the institution are severely constrained and perpetuate inherently exploitative power dynamics between academic institutions and communities.

## Introduction

Traditionally, youth education has avoided teaching hard truths about the history of enslavement, genocide, institutionalization, forced sterilization and other forms of sexist, ableist, and racialized realities of our past, often excluding communities that it tries to assist in the name of Equity, Diversity and Inclusion (EDI). This culture of exclusion has not escaped our academic institutions despite the recent surge in awareness around EDI issues. Academic institutions have been historically structured such that the voice of the academic is centered and amplified by itself—an echo chamber on what we often call “University Island” (Reingewertz and Lutmar, 2018; Williams, 2019). For example, while working collaboratively with community organizations, it is often mandatory to utilize the university academic calendar for funding, and institutional requirements generally direct the forms of programming, access, participant availability, frequency, and the scope of work. Due to the gender, race, ability, and other inequities found in academic circles (Freund et al., 2016; Cole, 2020), centering of academic voices can directly lead to the perpetuation of White supremacist ideologies and systems in well-meaning community engagement. These ideologies are found across the entirety of education, from K-12 to higher-ed. In today’s political climate more than ever, community engagement and EDI initiatives are being openly challenged by many conservative organizations (Yancy, 2018; Liu et al., 2021; López et al., 2021). Those intended to benefit from these efforts are excluded, marginalized, and silenced. Even with recent pushes towards more inclusive education models, structural inequities are resoundingly clear.

Historically, many institutional attempts to address root causes of structural and systemic inequities have been well-crafted statements without concrete action (Gilliam et al., 2021). Recently, there has been fervent resistance to such performative allyship (Coley and Holly, 2021). We must transition from overgeneralizing and diluting issues to identifying specific problems and recognizing the impact that we as academics have in addressing complex problems throughout our society. When speaking on community engagement work in the Science, Technology, Engineering, Agriculture, and Mathematics (STEAM) fields, there is a tendency to believe that “harder” sciences, including neuroscience, operate outside of the need for community voices and ways of knowing, and that this academic, “professional” centering is the only way to assure accuracy (Gilliam et al., 2021). If academics continue to be the only ones funded to pursue research questions, the results are often missed opportunities for communities, leading to disengagement and disenfranchisement (Gilliam et al., 2021).

Immediate changes are needed to shift the institutional climate to open dialogues, continuous education, and feedback at every level. With these issues in mind, the University of Minnesota (UMN) launched the Masonic Institute for the Developing Brain (MIDB) in 2021 to showcase how social change drives institutional reform. MIDB is a community-centered institution with an interdisciplinary clinical and community research enterprise that invests time to recognize and amplify the voices of community members and leaders in the design and implementation of our facilities, research questions, and clinical care. We aim to co-create knowledge that is accessible and trusted, and promotes healthy brain development and wellness. The unique collaborative MIDB approach is founded by its strategic research service hubs. These service hubs are actively engaged across departments to accelerate discovery, facilitate integration, and identify opportunities for community services and public policy by listening more than speaking, taking risks, and disrupting and rebuilding. The UMN is 171 years old, and has >50,000 students and >20,000 staff across 19 colleges. Building a collaborative “Community-first” culture within MIDB as part of this historic institutional backdrop is difficult and will be a long road. Here we describe our approach toward investment and building an inclusive environment to accelerate our impact on our community.

## Our Approach to Establishing Community Engagement

We believe that de-centering the dominant voice of academia involves recognizing that knowledge is located in many places, and that diverse ways of knowing will lead to better outcomes for research, clinical care, education, policies, and overall community wellbeing. With these guiding principles, the MIDB Community Engagement and Education (CEEd) Core was formed to create a culture of interactive community engagement, build strong reciprocal community connections, and collaboratively create infrastructure to foster bidirectional benefits.

### The listening model to co-create programming and infrastructure

The CEEd Core works alongside the community and elevates their voices. Rather than offering a menu of services asking the community to select from a predetermined list of programs, we formed programming based on the community’s expressed needs *via* direct and continuous engagement. To successfully co-create programming and infrastructure, the CEEd Core heavily utilized and adapted the principles of Heffner and Zandee’s work to create our listening model—a practice that initiates relationships with the community by first being present, listening, and acknowledging community voices without mention of any academia-originated programming (Heffner et al., 2003). For the first year of the CEEd Core’s inception, the Director, Dr. Anita Randolph, met with over 300 community organizations, attended dozens of community events, and volunteered throughout the community. Dr. Randolph networked with numerous diverse community groups, primarily grassroots organizations that dealt with equity and social justice work, but also larger nonprofits that worked in public health, brain development, and policy fields. There was a purposeful effort to talk to as many people on the ground as possible, not solely targeting upper leadership, to get a grasp for pressing issues in the community. This included individuals not representing a formal organization. By being a trusted community supporter first, and a faculty member second, Dr. Randolph was able to learn the community’s priorities, successes, concerns, needs, stakeholders, and what community members know as effective approaches for change. Only after multiple events and recognition in the community as a familiar face did Dr. Randolph introduce the CEEd Core and its goals, request feedback on existing programs, and propose a collaboration to form new community programming. It is important to stress that the introduction of the CEEd Core came after a request from community leaders. This approach to relationship building has been imperative in fostering reciprocal communication with the communities surrounding MIDB and establishing confidence that the CEEd Core values community feedback. Listening in this way has uncovered community needs in specific focus areas: mental health, addiction, programming to expose youth to STEAM careers, food sustainability, the impact of nutrition on neurodevelopment, and programming to demystify healthy brain development.

Community building relies on trusting the knowledge that people hold about their community. For example, during the design process of the MIDB building, community members and leaders were heavily involved, providing feedback on color themes throughout the building, wall textures, room signage, languages, accessibility features, and artwork. During our focus groups, Indigenous community members relayed that owls create an uncomfortable environment, which led to their removal from the artwork in the clinic. Additionally, focus groups including people with intellectual and developmental disabilities and their families resulted in the addition of several building features such as sit-to-stand adjustable tables in the conference and meeting rooms, adult changing tables in the restrooms, and adjustable lighting in common meeting spaces.

In addition to establishing infrastructure to create a more accessible, inclusive clinical environment, mistrust of research resonated throughout every conversation with the community. Although research is a fundamental step toward reducing disparities, it is often conducted “on” communities rather than “with” communities. In many cases, this has led to general distrust and, worse, total disengagement from research and clinical trial opportunities. Based on community feedback, it became evident that much of their mistrust towards research at the University stemmed from the lack of formal community-engaged research training of the UMN scientific community. From interviews with scientists in the neurosciences and brain-focused fields conducting community-engaged research and clinical trials, it was clear that researchers are often ill-prepared to be on the ground with community members and are unclear of basic community-engaged principles (e.g., appropriate use of community-engaged research methods, ethical practices, bidirectional community-institution benefits, etc.). This has resulted in burned bridges between the University and its community partners, because scientists often used top-down practices common in academic settings, did not share decision-making appropriately with the community, and failed to acknowledge the community’s knowledge and expertise. The lack of methodological training in community-engaged research has caused harm within the community, misappropriation of community members’ time, trauma, extraction of their knowledge, and generational mistrust of University researchers in general, limiting outcomes for both the scientific community and the greater community as a whole.

This problematic dynamic has been further complicated by recent National Institutes of Health guidelines requiring the creation of novel community-engagement cores that serve a given grant. What this means is that institutions are under newfound pressure to begin research efforts that involve the community’s active participation. This has created another issue, as these historically underfunded engagement programs do not have the resources, capacity, or infrastructure in place to handle this new push to support basic research scientists. The CEEd Core has tackled this complex issue with a multipronged approach. First, the CEEd Core has prioritized time, funding, and effort into producing training modules for students, staff, faculty, and community researchers to gain knowledge in community-engaged research methods, equitable and sustainable partnerships, evaluation, dissemination, and best practices of ethical exits to minimize harm to communities after the completion of the project. Our community-engaged research training modules are community-informed, utilizing both external experts and community leaders as co-facilitators and co-owners of the materials. The infrastructure of the community-engaged training modules was built to allow ongoing, yearly training that is both reactive (i.e., the immediacy of a need dictates the order of the module development) and community-informed (i.e., the modules are built to be used by an assortment of stakeholders). The goals of these modules are to produce a new generation of community-informed researchers who will utilize the concepts of community-engaged research, minimize harm when working with the community, and produce community-engaged scholarly products aligned with their basic science research.

Additionally, the CEEd Core has adopted the practice of centering research priorities identified by the community rather than only topics selected by research teams at the University, training teams on how to share research findings with the community in real time, and collaborate with the greater MIDB system to create solutions to decrease the years it takes for research findings to be integrated into clinical practice to ensure tangible change in the community.

The CEEd Core also founded the development of the Neuroscience Opportunities for Discovery and Equity (NODE), a centralized arena for the development of neuroscience-focused engagement programs across 10 separate departments at UMN. NODE’s collaborative nature prevents silos between engagement-focused groups across the University to reduce duplicated efforts, cost, and staffing barriers.

### Neuroscience opportunities for diversity and equity (NODE)

In our experience, the effectiveness of community engagement is difficult to quantify; trust is observed in subtle changes in community interactions. For example, establishment of trust may be represented by unsolicited invitations to community events in informal safe spaces that include youth and elders who are typically shielded from formal discussions. A community member’s receptiveness to services and perspective may be represented by spontaneous communication *via* text/phone to request information or to share an experience or just to be heard. A community’s willingness for collaboration has been signaled by direct communication with community leaders in sacred places not intended for outsiders. Waiting patiently for permission to engage with the community takes time, which is not valued or easily translated into community-engaged scholarly products.

With this in mind, Dr. Randolph interfaced with multiple departments and programs at UMN to learn the many challenges of community-engaged research within the University system, identified silos to dismantle to enhance our work, and gauged receptiveness to developing community-engaged research infrastructure. With a vision of increasing capacity through collaboration, a group called the Neuroscience Opportunities for Discovery and Equity (NODE) was formed to form a pool of shared resources to enhance engagement with the community across 10 different departments.

Through bimonthly meetings, NODE members have been able to request help in a variety of ways. While some departments have a lot of funding and no staff, they are now able to ask for help from others’ research assistants, student workers, and volunteer networks from those who have adequate staffing or gaps in their engagement calendars. This imitable model can help departments overcome challenges associated with limited time, funding, and staffing to reduce burn-out, and ensure tangible solutions that improve people’s lives in the community while inspiring the next generation of underrepresented learners to become scientists.

In its first year, NODE members worked together to fund comprehensive validated surveys and personalized evaluation services for many of the engagement projects across these departments. Members have been able to utilize the evaluation services in order to apply for private donors and NIH grants to secure a future for their group. With that essential piece covered, translating community engagement into scholarly work is now a much more affordable and easier task. NODE was also able to secure thousands of dollars worth of engagement supplies, ranging from multiple brain and spinal cord models, plastinated human brains, a 3D printer, and various engagement games and interactive activities. NODE works with a goal of collaborating on engagement events and grant applications. By combining different departmental missions and engagement work into cohesive, fundable projects, this small shift has opened up the possibility of well-funded, researched collaborative efforts focused on community engagement.

## Leveraging The “Community First” Engagement Infrastructure to Diversify The Steam Workforce

As noted above, one outcome of the established CEEd Core engagement models was learning about the community’s desire for programming to expose youth to STEAM research and careers. To diversify STEAM, we must embrace “variability”—our diversity—and provide access to this pursuit to all of the talents that exist in our society. Ironically, in the sciences, our ability to proportionally value the importance of this principle has been limited. Although neuroscience is considered one of the fastest growing disciplines, the lack of URMs and/or disabled scientists has led to a lack of diversity in research studies, inadequate representation in higher academic positions, limited scholarly perspectives, and the perpetuation of inequities in the science fields (Bertolero et al., 2020; Jones-London, 2020).

Recruiting, training and retaining a diverse pool of highly skilled individuals in neuroscience is imperative for maximizing our investments and potential in research and education. In the US, despite many national efforts, URMs and/or those with disabilities continue to be underrepresented as neuroscience undergraduates, trainees, faculty, and in the overall research workforce. According to the Society for Neuroscience, 23% of students enrolling in neuroscience Ph.D. programs and 14% of Ph.D. awardees in 2016/2017 were students from underrepresented backgrounds and 15% of postdoctoral trainees and 8% of program faculty identified with underrepresented backgrounds (Society for Neuroscience, 2017). Given that the 2020 US Census reported that 42.1% of the US population identifies as coming from an underrepresented backgrounds (Berry-James et al., 2020), these statistics indicate that there is an unmet need for innovative programs that foster recruitment and retention of URMs and/or disabled students in the neuroscience workforce, in order to better reflect the broader population that neuroscience research seeks to benefit.

Advances in health care, education, technology, and other enhancements to our society that deeply touch our everyday lives will not come with a homogeneity of ideas, education, experience, and culture. The CEEd Core’s in-depth interviews with community members confirm communities of color are distrustful of academic and health-centered institutions and their engagement practices. Not only does such disengagement prevent underrepresented youth from pursuing STEAM degrees and gaining economic earning power to contribute back to their community, but the lack of representation in healthcare and technology fields exacerbates URM communities’ distrust of healthcare professionals. This is a significant issue in Minnesota as the URM population grows without a concomitant increase in the state’s workforce (Flaherty, 2021; Khalid and Snyder, 2021). Consequently, the participation of URMs in STEAM is critical to address the growing health, education, and human service needs of our increasingly diverse population. To help close this gap, it is imperative to begin engaging with URM students in K-12, undergraduate, and graduate school, as well as their families, to increase STEAM participation and develop the next generation of URM scientists. In the Supplementary Materials, we describe four such programs including the Young Scientists (YS) Program, the Youth Engaged with Science (YES!) Program, NextGen Psych Scholars Program (NPSP), and the MN Leadership Education in Neurodevelopmental Disabilities (MN LEND) Program.

## Discussion

The endemic issues of structural racism, ableism, sexism, and other inequities cannot be addressed by changes in policies and practices alone: change will require direct action and work in the trenches with our communities. To de-center the academic voice, we, the academic community, must move away from the authoritarian approach of creating, disseminating, and teaching knowledge. Although the efforts of MIDB are still in its early stages, the work continues to grow and shape itself through directly listening to community members, acting and reacting, and pursuing a mission of diversity and representation in programming, infrastructure, and staffing. Within MIDB and the work of the many partners listed in this article, options for potentially transformative research and practices are able to be explored to help address systemic concerns that continue to haunt institutions. Although beneficial, continuing to elevate diverse racial, ethnic, and linguistic communities, families, and youth through participation in STEAM fields will not clean or sterilize past atrocities. As we learn from our past to move forward, we must create solutions. Our various efforts are focused on increasing the presence of and support for a more diverse body of scholars and scholarship in science and other fields that continue to only have a sprinkling of diversity. Our efforts involve connecting with community members, families, schools, and scholars. These connections must be nurtured through relationships, trust, and the recognition of each other’s humanity. Most importantly, nurturing and building trust takes time and must be supported by leadership to yield fruitful change. Only then can we walk in our truth of being with and for the community.

## Data Availability Statement

The original contributions presented in the study are included in the article/Supplementary material, further inquiries can be directed to the corresponding author.

## Author Contributions

AR is the first author and contributed heavily throughout this article. AHen contributed to writing and editing throughout. AM contributed to writing throughout the supplements of this publication. RS contributed to the writing and concept of this article. MD and MK contributed to writing throughout the supplements. JE contributed to writing throughout the introduction and conclusion, and editing. DF contributed to writing, editing, and conceptual support. All authors contributed to the article and approved the submitted version.
