# Supplementary material for: Creating a sustainable action-oriented engagement infrastructure—a UMN-MIDB perspective
Source: Front Integr Neurosci. 2022 Dec 15;16:1060896. doi: 10.3389/fnint.2022.1060896 (PMC9797530; doi:10.3389/fnint.2022.1060896)
Supplement: Supplementary file 1 [file Data_Sheet_1.docx]

Supplementary Materials

## The Youth Engaged in Science (YES!) Program

Recent research finds that, due to a variety of factors, the percentage of URM students who start in STEAM majors and go on to graduate with STEAM degrees is significantly less than the percentage of their White peers ([(Riegle-Crumb, King, and Irizarry 2019)](https://paperpile.com/c/gdUSK7/WsQwb). The authors attribute the disparities in URM completing STEAM degrees to a culture in STEAM fields which fosters competitive, “exclusionary spaces where URM students must essentially prove that they deserve to stay” [(Beasley 2011; Correll, Seymour, and Hewitt 1997)](https://paperpile.com/c/gdUSK7/HWv2G+SEeCK). Beasley and Fischer proposed that the adverse effects of this type of high-stress environment can be mitigated by exposing URM students to role models who are also URMs in STEAM. This type of modeling, exposure, and empowerment is the goal of the Youth Engaged in Science program! (YES!) Program.

One of the goals of the Community Engaged and Education (CEEd) Core is to build a diverse, equitable environment where all can thrive and excel. In walking in that mission, we are driven to create programming to increase the representation of underrepresented minorities (URMs) in the science, technology, engineering, agriculture, and mathematics (STEAM) fields. Increasing the diversity in STEAM will enrich the scientific community and its ability to serve an increasingly diverse and rapidly changing population, create opportunities for communication between professionals of varied backgrounds, and increase the amount of unique, creative ideas, and approaches to problem-solving. The presence of diversity in the workplace encourages students of all backgrounds to not only pursue scientific careers but also to be able to successfully navigate the road to achieving them because exposure to URM scientists and clinicians can open doors to mentorship and modeling of a career trajectory.

The YES! program aims to increase the disproportionately low number of underrepresented minorities in STEAM fields. Our approach to addressing these issues is to offer exciting and relevant science educational programs to middle- and high-school students who are from groups that are underrepresented in STEAM. These groups include people who identify as Black/African American, Hispanic/Latino(a), American Indian/Alaska Native, Native Hawaiian & Pacific Islander, disabled, and or those from disadvantaged backgrounds. We bring these students into contact with URM clinicians and researchers to help URM students realize they belong and are welcome in the STEAM fields. Additionally, the YES! program offers science education programs and activities to middle- and high-school students, including neuroscience workshops at schools, educational tours of the University of Minnesota (UMN) research laboratories and centers, interactive demonstrations of scientific research and clinical practice applications at large-scale community outreach events, and panels highlighting various topics to enhance the applications of potential URM students. Pre- and post-surveys with program participants reveal that a majority of students want to learn more about careers in the science field and more about their brains after participating in our activities. We believe that by engaging in hands-on science activities and connecting with URM scientists, students are cultivating increased self-efficacy and inspiration.

## The Young Scientists (YS) Program

The Young Scientists program aims to cultivate positive and engaged science identities in middle school and high school students from traditionally underserved communities in the Twin Cities area. The YS program connects graduate student mentors from ICD, Psychology, and Educational Psychology with middle and secondary school students in a weekly after-school program where small groups collaborate and develop research projects in the general fields of developmental psychology and neuroscience. The YS program emphasizes relationships and process, and engages in the kind of collaborative teamwork that all scientific work depends upon. By building relationships between young scholars and graduate student mentors, YS scholars gain the tools of scientific inquiry and apply them as they ask their own original questions and design studies to answer those questions. At the end of the year, students in the YS program have both conducted their own primary research projects and are poised to present their research in a poster presentation, either at a professional scientific conference (e.g., Building Pipelines: Engaging Youth in Science, SRCD2021) or a local poster symposium at MIDB. Like the name of the program implies, a central premise of the YS program is to give participating students a full-blown, first-hand experience of walking the path of a young scientist.

One of the partner schools for Young Scientists is Ascension Catholic School in North Minneapolis. For Ascension’s Principal, Mr. Benito Matias, there’s a well-known achievement or opportunity gap in education for students of color in Minneapolis but there’s also a “belief gap:” “The belief gap has to do with scholars believing in their own ability to achieve, and the adults around them believing in their ability to achieve,” says Matias. The Young Scientist program is intentionally designed to support scholars’ beliefs in their own abilities and identities as scientists, which is mirrored by every graduate student mentor who believes they can do it.

## Students from Young Scientists spend the first part of the year spending quality time with graduate student mentors and forming project groups, as they learn about different approaches that scientists take to study children and development. By beginning in this way, the focus of YS consistently rests upon a process of engagement and relationship-building. As the program moves into the second half of the year, scholars in project groups begin to form questions, make decisions, and design a study that they can execute in the school. YS projects reflect a range of topics: learning and memory, number cognition, trust decisions, resource allocation and more. For the YS scholars, one really fun component of conducting the research they design is interacting with younger students at Ascension as they collect participant data from them. In turn, the younger children at Ascension enjoy having the chance to participate in a study that was designed by older scholars they often know and recognize. In all of these ways, the YS program recognizes that knowledge is located in many places, and that diverse ways of creating knowledge will lead to better outcomes for students and everyone who participates.

## NextGen Psych Scholars Program

Among the STEAM fields, the psychological sciences has long struggled to recruit and retain talented diverse scholars, with one of the biggest bottlenecks in the training pipeline occurring during the transition from undergraduate to graduate school [(Borrego 2018; DeJoseph, M. L. , Carosella, K. , & The NextGen Lead Mentors, n.d.)](https://paperpile.com/c/gdUSK7/LJVN+XwUR). The NextGen Psych Scholars Program (NPSP; [nextgenpsychscholars.com](http://nextgenpsychscholars.com)), founded in 2020 by UMN psychology PhD students Meriah DeJoseph and Kate Carosella, was created to respond and overcome this training bottleneck. NPSP is a one-year(+) *virtual peer-to-peer mentorship program* for underrepresented undergraduates and post-baccalaureates interested in applying to psychology Ph.D. programs. Now entering its third year, NPSP has served over 550 national and international undergraduates and post-baccalaureates (250+ in year 1; 300+ in year 2) and included graduate student and postdoctoral mentors (80+ in year 1; 200 in year 2) from across the United States and abroad. Through a combination of regular virtual panels, workshops, online correspondence, and monthly one-on-one mentorship meetings, NPSP focuses on creating and sustaining strong mentoring relationships, demystifying the hidden curriculum of academia [(Calarco 2020)](https://paperpile.com/c/gdUSK7/5RJlo), and building a supportive community of diverse scholars that advocate for themselves and others with the goal of innovating academia from the bottom-up.

The primary programming across the year is created and led by 10-12 “lead” mentors, who are URM Ph.D. students. Meriah DeJoseph, the NPSP Founder and first-generation college graduate who benefited from peer mentors while pursuing higher education, believes that the unique perspectives that URM Ph.D. trainees offer NPSP programming is critical to fostering a greater sense of belonging within NPSP and the broader academic community. Connecting with the next generation of diverse psychology scholars on a more human level–where lived experience and context are embedded throughout–may be a necessary first step towards diversifying academia from the bottom-up. Such connections are often formed most easily among peers who can provide mentorship that inherently feels less intimidating [(McCarthy et al. 2012)](https://paperpile.com/c/gdUSK7/b1XWR). Indeed, such shared lived experience among peers fosters a trusting and safe mentorship relationship, which is necessary for ensuring the success of the diverse prospective students psychology programs hope to recruit and retain.

Given NPSP’s reliance on the perspectives and leadership offered by lead mentors, a core component is its commitment to amplifying, acknowledging, and properly incentivizing the voices of historically underrepresented young scholars in the psychological sciences. Thus, a particularly unique feature of NPSP is that acquired university funding goes towards providing yearly consultant payments to its lead mentors, in addition to smaller payments to 50 UMN graduate student mentors who mentor at least one mentee and participate in panels as needed. NPSP continues to grow and is partnering with academic societies and other university psychology Ph.D. departments to ensure additional graduate mentors from across participating psychology Ph.D. programs are paid for their time and mentorship. A growing collaboration with MIDB and Dr. Anita Randolph will allow NPSP to expand and enhance its current model for training mentors in socioculturally-sensitive mentorship practices.

Collectively, NPSP aims to lift up the next generation of diverse scholars in the psychological sciences. These are the scholars who will become the leaders our field needs to produce long-term positive change, bringing long overdue representation and connection to marginalized communities through scientists who themselves have grown up in those communities. Doing so will ultimately improve our science and resulting community impact by enhancing the questions we ask, the methods we use to capture lived experiences, and ensure the conclusions we draw are rooted in context and integrity.

# MN Leadership Education in Neurodevelopmental Disabilities (MNLEND)

The Institute on Community Integration (ICI) at MIDB specializes in policies and practices that affect children, youth, and adults with disabilities, and those receiving educational support. The ICI ensures that people with disabilities are valued by, included in, and contribute to their communities of choice throughout their lifetime by developing new leaders from racially, ethnically, and linguistically diverse backgrounds through the Minnesota Leadership Education in Neurodevelopmental and Related Disabilities (MNLEND) Program. MNLEND is an interdisciplinary graduate level training program focused on developing leaders across many academic fields who will support children, youth and adults with neurodevelopmental disabilities, and their families. A unique feature of the MNLEND program is that academic fellows learn side by side with community fellows (including family members, persons with lived experience of NDD and current practitioners) through a 9 - 12 month cohort model. Each year 30-35 fellows are recruited, selected, and trained in this model using an accessible curriculum to work on community identified and driven projects. These projects are sometimes research, evaluation, program development, practice or policy focused.

There are numerous strategies used to ensure that MNLEND is able to recruit and retain diverse fellows in its program. MNLEND faculty work with community organizations to identify their needs and connect them with fellows for future employment, this often results in them referring new fellows to the program. Over the years we have built a strong alumni program and we hire diverse alumni to mentor fellows and to offer fellows site visit, clinical visit and project opportunities within the organizations in which they work. This reciprocity has built trusting relationships over time and increased the community network and reach of the program.

On the university campus, we collaborate with the TRIO and McNair Scholars programs. TRIO is a federally funded program that supports students from disadvantaged and underrepresented backgrounds as they pursue an undergraduate college degree. TRIO student supports include: academic tutoring, personal counseling, mentoring, financial guidance, and other supports necessary for educational access and retention. The UMN TRIO program is located in the College of Education and Human Development and is focused specifically on those students who are low income, first generation and have disabilities. The McNair Scholars program seeks to increase doctoral program application, matriculation and degree attainment by underrepresented and first-generation students. MNLEND fellows from diverse racial, ethnic, linguistic and disability backgrounds mentor students in these programs and help them to better understand career options in neurodevelopmental disabilities and the related graduate level education requirements. This increases awareness for TRIO and McNair students about many different careers and the various roles professionals can play in supporting individuals with neurodevelopmental disabilities and their families and offers support to them if they choose to explore these careers further. In addition to MNLEND fellows meeting with McNair and TRIO students in their programs, the students are invited to attend MNLEND didactic sessions and, as desired, events, organization, clinical and research observations. This reciprocal relationship sometimes leads to students deciding to enter graduate programs related to NDD and later applying to become MNLEND fellows once they are engaged in a graduate program in an approved academic discipline.

**References:**

[Beasley, M. 2011. “Opting out: Losing the Potential of Americas Young.” Black.](http://paperpile.com/b/gdUSK7/HWv2G)

[Borrego, Joaquín, Jr. 2018. “It Takes a Village for Meaningful and Sustainable Change in Diversifying Psychology.” *Training and Education in Professional Psychology* 12 (4): 297–300.](http://paperpile.com/b/gdUSK7/LJVN)

[Calarco, Jessica Mccrory. 2020. *A Field Guide to Grad School: Uncovering the Hidden Curriculum*. Princeton University Press.](http://paperpile.com/b/gdUSK7/5RJlo)

[Correll, Shelley J., Elaine Seymour, and Nancy M. Hewitt. 1997. “Talking about Leaving: Why Undergraduates Leave the Sciences.” *Contemporary Sociology*. https://doi.org/](http://paperpile.com/b/gdUSK7/SEeCK)[10.2307/2655673](http://dx.doi.org/10.2307/2655673)[.](http://paperpile.com/b/gdUSK7/SEeCK)

[DeJoseph, M. L. , Carosella, K. , & The NextGen Lead Mentors. n.d. “Diversifying the Psychological Sciences through a Peer-to-Peer Mentorship Model: Insights and Recommendations from the NextGen Psych Scholars Program.”](http://paperpile.com/b/gdUSK7/XwUR)

[McCarthy, Sherri, K. Laurie Dickson, Jacquelyn Cranney, Annie Trapp, and Victor Karandashev. 2012. “Peer-to-Peer Mentoring: An Embedded Model to Support the Transition of First Year Psychology Students.” In *Teaching Psychology around the World: Volume 3*, edited by Sherri McCarthy, K. Laurie Dickson, Jacquelyn Cranney, Annie Trapp, and Victor Karandashev, 18. Newcastle upon Tyne, United Kingdom: Cambridge Scholars Publishing.](http://paperpile.com/b/gdUSK7/b1XWR)

[Riegle-Crumb, Catherine, Barbara King, and Yasmiyn Irizarry. 2019. “Does STEM Stand Out? Examining Racial/Ethnic Gaps in Persistence Across Postsecondary Fields.” *Educational Researcher*  48 (3): 133–44.](http://paperpile.com/b/gdUSK7/WsQwb)
